# *Sechium edule* (Jacq.) Swartz, a New Cultivar with Antiproliferative Potential in a Human Cervical Cancer HeLa Cell Line

**DOI:** 10.3390/nu9080798

**Published:** 2017-07-25

**Authors:** Sandra Salazar-Aguilar, Lucero del Mar Ruiz-Posadas, Jorge Cadena-Iñiguez, Marcos Soto-Hernández, Edelmiro Santiago-Osorio, Itzen Aguiñiga-Sánchez, Ana Rocío Rivera-Martínez, Juan Francisco Aguirre-Medina

**Affiliations:** 1 Postgraduate College, Campus Montecillo, Km 36.5 Mexico-Texcoco Highway, C.P. 56230 Texcoco, Mexico; saguilars@colpos.mx (S.S.-A.); msoto@colpos.mx (M.S.-H.); 2 Interdisciplinary Research Group of *Sechium edule* in Mexico (GISeM), Texcoco, Agustín Melgar 10 Street, C.P. 56153 Texcoco, Mexico; jocadena@colpos.mx (J.C.-I.); edelmiro@unam.mx (E.S.-O.); liberitzen@yahoo.com.mx (I.A.-S.); anarocio.rivera@hotmail.com (A.R.R.-M.); juanf56@prodigy.net.mx (J.F.A.-M.); 3 Postgraduate College, Campus San Luis Potosí, Iturbide No. 73 Street, Salinas de Hidalgo, C.P. 78600 San Luis Potosí, Mexico; 4 FES Zaragoza, National Autonomous University of Mexico, Batalla 5 de mayo esq. Fuerte de Loreto, Col. Ejército de Oriente, C.P. 09230 Mexico City, Mexico; 5 School of Higher Education of Agricultural Science, Autonomous University of Chiapas, Junction Costera Higway and Pueblo de Huehuetán, Huehuetán, C.P. 30660 Chiapas, Mexico

**Keywords:** cancer, Perla Negra, chayote, biological activity

## Abstract

The *Sechium edule* Perla Negra cultivar is a recently-obtained biological material whose progenitors are *S. edule* var. *nigrum minor* and *S. edule* var. *amarus silvestrys,* the latter of which has been reported to have antiproliferative activity against the HeLa P-388 and L-929 cancer cell lines. The present study aimed to determine if the methanolic extract of the fruit of the Perla Negra cultivar had the same biological activity. The methanolic extract was phytochemically characterized by thin layer chromatography (TLC) and column chromatography (CC), identifying the terpenes and flavonoids. The compounds identified via high performance liquid chromatography (HPLC) were Cucurbitacins B, D, E, and I for the terpene fractions, and Rutin, Phlorizidin, Myricetin, Quercetin, Naringenin, Phloretin, Apigenin, and Galangin for the flavonoid fractions). Biological activity was evaluated with different concentrations of the methanolic extract in the HeLa cell line and normal lymphocytes. The methanolic extract inhibited the proliferation of HeLa cells (IC_50_ 1.85 µg·mL^−1^), but the lymphocytes were affected by the extract (IC_50_ 30.04 µg·mL^−1^). Some fractions, and the pool of all of them, showed inhibition higher than 80% at a concentration of 2.11 µg·mL^−1^. Therefore, the biological effect shown by the methanolic extract of the Perla Negra has some specificity in inhibiting tumor cells and not normal cells; an unusual feature among molecules investigated as potential biomedical agents.

## 1. Introduction

Chayote (*Sechium edule* (Jacq.) Swartz) (Cucurbitaceae) is cultivated on the American continent from Mexico to South America and the Antilles [1]. In Mexico, the states of Chiapas, Oaxaca, and Veracruz present the greatest genetic diversity worldwide; in particular, Veracruz is the state with the richest biodiversity of this species [2,3]. A wide variety of shapes, colours, flavours, and textures can be observed in the fruit.

The importance of the cultivated *S. edule* biological varieties include dietary and socioeconomic aspects from the generation of profits in export markets [4]. In the pharmacological area, different studies have reported the use of parts of the plant to treat different illnesses, such as low blood pressure, and it is also used as a vasodilator [5,6]. The roots, stems, and leaves are used as antimicrobial agents [7], and the leaves with seeds are used as antioxidants [8]. On the other hand, anti-inflammatory and cardiotonic activity of the fruit has been reported [9,10]. Its use in the treatment of arteriosclerosis has also been reported [11], as well as in depressing the central nervous system and treating epilepsy [12].

These studies show promising uses of *S. edule*; however, the biological variant was not specified in any of them. This situation is important if the broad morphological and biochemical diversity contained in the intra-specific variation of the species is considered [13,14], because the type and content of their secondary metabolites is very different in a variety of groups, which has repercussions on the biological activities that they may present [14].

The cytotoxicity of eight variants of the fruit from *Sechium edule* on different cancer cell lines has been reported [15], highlighting that varietal groups (*albus dulcis, albus levis, amarus silvestrys, nigrum spinosum*, and *nigrum xalapensis*) inhibit P-388 (mouse monocytic leukaemia) and L-929 (mouse fibrosarcoma lung) cells [16], while *nigrum spinosum* showed an antiproliferative effect against HeLa (human cervical cancer cell line) and L-929 [17] cells, while inhibiting the viability of WEHI-3 (mouse myelomonocytic leukaemic cell line) cells [18]. Additionally, *albus levis, albus dulcis, albus minor, amarus silvestrys, nigrum spinosum, nigrum levis, nigrum xalapensis* and *virens levis* had antiproliferative effects on L-929, HeLa, and P-388 cells [16]. The main secondary metabolites identified and related to biological activity were alkaloids, sterols, saponins, triterpenes [9,15,19], saturated fatty acids esters [17], glycosylated flavonoids [19,20], and tannins [18].

From this perspective, it is relevant to perform in parallel bioprospection studies of genetic improvement programmes in *Sechium edule,* so that the range of outstanding genotypes for pharmacological applications that are easy and safe to use at lower concentrations can be broadened, given the plasticity of the environmental adaptation of the species [13,21,22].

The *Sechium edule* Perla Negra cultivar is a new genotype obtained by interspecific crossing in the field, and its progenitors are *S. edule* var. *nigrum minor* and *S. edule* var. *amarus silvestrys*. The aim of this paper was to determine the metabolite content of the methanolic extract of the fruit of this cultivar, and the potential antiproliferative effect on HeLa cells, starting from the premise that different genetic lineages can have different effects and be used as a source for complementary medicine.

## 2. Materials and Methods

**Plant material:** Fruits of the Perla Negra cultivar, whose genetic lineage comes from a process of improvement [23,24,25] of biological variants of the varietal group *Sechium edule var*. *nigrum minor*, were crossed with the group *Sechium edule var. amarus silvestrys*. Once the cultivar was stabilized, the fruits were collected by the Interdisciplinary Research Group of *Sechium edule* in Mexico, A. C. (GISeM) in the Bank of Germoplasm (19°08′48″ N, 97°57′00″ E). A voucher specimen 631-12 remains in the same place.

**Extraction procedure**: Fruits of the Perla Negra cultivar were cut into small pieces, dried at 45 °C to 10% moisture and ground to a standardized particle size of 2 mm [15]. Next, 1013 g of plant material was extracted in a batch with methanol (99.8%, ACS, Merck, Darmstadt, Germany) for 48 h at room temperature (20 ± 2 °C), and the resultant alcoholic extract was filtered with paper 12 times, renewing the solvent until the macerated product was not coloured. Then, the solvent was evaporated at 50 °C under reduced pressure (Buchi Rotavapor R-114, Flawil Switzerland), until a crude extract, without organic solvent, was obtained [26,27,28]. A stock solution was made with 71.2 mg of crude extract solubilized with 300 µL of ethanol (96%) and 700 µL of phosphate buffered saline (PBS; Sigma, St. Louis, MO, USA), and corresponding dilutions were made. The obtained fractions were evaluated at 2.11 µg·mL^−1^ (IC_50_ statistical), a stock solution was made with 5 mg of each fraction and solubilized with 100 µL of ethanol (96%) and 900 µL of PBS, and then 40 µL was taken of the mixture and solubilized with 960 µL of PBS to have the concentration defined. The pool took 2.1 µL of each fraction (42 µL), and 958 µL of PBS. The three mixtures were centrifuged at 500 g for 5 min (Hermle-Labortechnik, Wehingen, Germany), and the supernatant was subsequently filtered and stored at 4 °C.

**Fractioning through Column Chromatography (CC)**: A sample of 10 g of the methanolic extract was employed, and a mobile extraction phase was applied to it with hexane at 100%. Later, a hexane:ethyl acetate mixture was gradually added in a decreasing decimal relation of 90:10 until reaching its inverse, 10:90, and ending with ethyl acetate at 100%. After the first stage, a second extraction was performed with ethyl acetate:methanol in the same decreasing decimal manner as the first, ending with methanol at 100% [17]; then, the sample was brought to reduced pressure at 45 °C to make it solvent-free.

**Identifying compounds by TLC**: Preliminary tests were performed by thin-layer chromatography (TLC) on silica gel plates (Merck G60 70-230 mesh, Darmstadt Germany) with a mobile phase and a specific chromogenic agent. For the terpene fraction, mixtures of solvents with different polarities were used, the first with hexane:ethyl acetate (5:5), the second with hexane:ethyl acetate (2:8), and the third with ethyl acetate:methanol (8:2). They were developed with the chromogenic agent 1% vanillin and 10% sulfuric acid (in ethanol) [29], including the standards of Cucurbitacin B, D, E, and I. For the flavonoid fraction, the mobile phase of ethyl acetate:formic acid:acetic acid:water was used in the 100:11:11:26 ratio, and as the chromogenic agent, a mixture of 2-aminoethyl diphenyl-borinate:polyethylene glycol (1% NP, 5% PEG) was used. The standards were Rutin, Phloridzin, Myricetin, Quercetin, Naringenin, Phloretin, Apigenin, and Galangin. The plates were observed with UV at 254 and 365 nm (Cole-Parmer 9818, Chicago, IL, USA) before they were developed [29].

**Identifying compounds with biological activity by HPLC**: The compounds that had biological activity were evaluated by high performance liquid chromatography (HPLC); for the terpene fraction, the conditions were (Symmetry Shield Column RP18 (4.6 × 250 mm), Isocratic Analysis, solvents water:methanol:acetonitrile (50:30:20); flow 1 mL min^−1^; pressure 179 bars; temperature 20 °C; injection volume: 20 µL; λ1: 235 nm, λ2: 254 nm; and analysis time 50 min). For the flavonoid fraction, the conditions were (Hypersyl ODS (125 × 40 mm) Hewlett Packard Column, solvents (a) H_2_O pH 2.5 with trifluoroacetic acid) (TFA) and (b) acetonitrile (ACN) with flow 1 mL·min^−1^, temperature 30 °C, injection volume: 15–20 µL; λ1: 254 nm, λ2: 316 nm, and analysis time 25 min). The standards were Rutin, Phlorizidin, Myricetin, Quercetin, Naringenin, Phloretin, Apigenin, and Galangin. Finally, the results determined the concentration of the compounds based on the injection volume and detected micrograms. The terpene fraction was taken as a reference concentration of 2 mg·mL^−1^, which was 40 mg·mL^−1^ for flavonoids.

**Cell culture**: The HeLa cervical cancer cell line was obtained from the American Type Culture Collection (ATCC) (Rockville, MD, USA). The HeLa cells and human lymphocytes obtained by peripheral blood were cultured in Petri dishes (Sarstedt AG & Co., Wiehl, Germany) with Iscove’s modified Dulbecco’s medium (IMDM) (Gibco-BRL, Carlsbad, CA, USA) supplemented with 10% foetal bovine serum (FBS) (Gibco-BRL, Carlsbad, CA, USA), 100 units/ml penicillin, and 100 μg·mL^−1^ streptomycin (Sigma-Aldrich, St. Louis, MO, USA). The cells were maintained in a humidified atmosphere with 5% CO_2_ at 37 °C, and the culture medium was changed every two days.

**Proliferation assay:** The effect of the *Sechium edule* Perla Negra cultivar on cell proliferation was determined by the crystal violet assay [30,31]. The cells, cultured in triplicate in 96-well plates, were HeLa cervical cancer cells (2 × 10^4^ cell·mL^−1^) and lymphocytes human cells (4 × 10^5^ cell·mL^−1^); they were treated for 72 h with the methanolic extract at different concentrations (1.25, 1.56, 1.85, 2.5, 5, and 10 µg·mL^−1^ obtained from stock solution) and the antineoplastic agent, Cisplatin™ (0.72 µg·mL^−1^). The cells were fixed with glutaraldehyde at 1% for one hour; subsequently, they were stained with crystal violet solution (Sigma, St. Louis, MO, USA) after 10% acetic acid. The absorbance of the cells was measured at 570 nm using a microplate reader (Spectra Tecan Image, Grödig, Austria). This experiment was performed in triplicate, the statistical analysis was performed to obtain the final values, and the mean inhibition concentration [IC_50_] was calculated by the linear regression equation.

**Statistical Analysis**: Results were taken from three independent experiments in triplicate (*n* = 9), and the data were expressed as the means ± standard error. The statistical analysis was performed through an analysis of variance (ANOVA) and Tukey tests, with statistical significance at *p* < 0.001, using Software IBM SPSS (version 20.IBM Corporation, Chicago, IL, USA).

## 3. Results

**Phytochemical characterization:** The metabolites identified in the plant extract of the Perla Negra cultivar were terpenes and flavonoids. The estimated yield from the plant sample of 1.13 kg was 7.48% after the extraction, which was equivalent to 78.302 g of raw methanol extract. When fractioned, it showed a composition of 27.53 g of terpenes distributed in 17 fractions and 32.85 g of flavonoids recorded in three fractions. The terpenes observed in the TLC plates were primarily related to glycosylated terpenes [32], as a function of the coloration and retention value (Rf) from dark blue, turquoise, coffee-coloured, brown, purple, dark green, to violet tones. The blue and red-violet tones were related to cucurbitacins (tetracyclic triterpenes) [29], which give a bitter flavour to the fruits [9,14]. Regarding flavonoids, a range of colours between magenta, orange, yellow, and violet was observed, which was related to the chemical groups of flavonols, flavones, and flavonones [29]. The magenta colour coincided with the standard for quercetin (Rf 0.95), the orange colour was related to the standard chlorogenic acid (Rf 0.5), and the yellow was associated with the routine standard (Rf 0.35).

**Figure 1:**
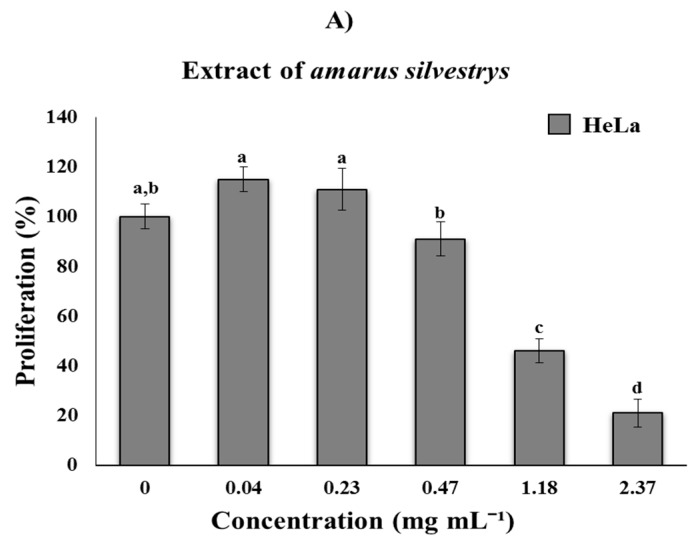

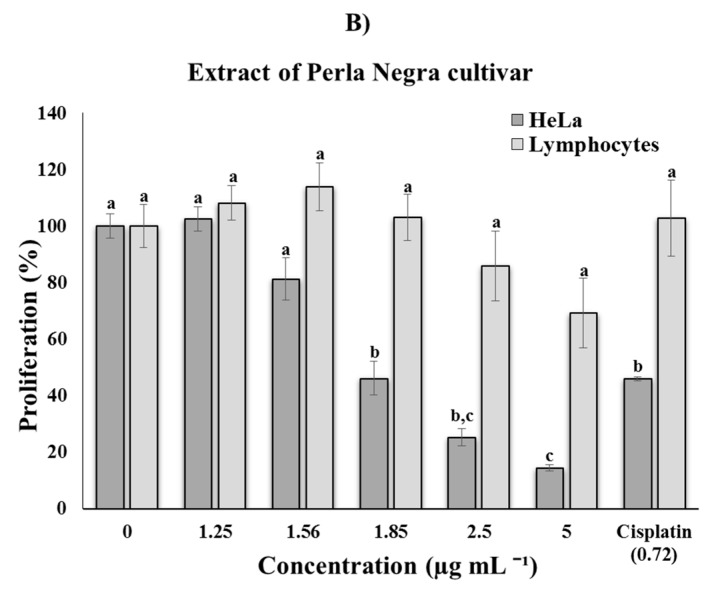
Proliferation assay. (**A**) Inhibitory effect of *amarus silvestrys* on HeLa cells; (**B**) HeLa cells and lymphocytes with the Perla Negra methanolic extract at different concentrations (0 µg·mL^−1^, 1.25 µg·mL^−1^, 1.56 µg·mL^−1^, 1.85 µg·mL^−1^, 2.5 µg·mL^−1^, and 5 µg·mL^−1^) and Cisplatin (0.72 µg·mL^−1^) for 72 h. Different letters indicate statistical significances 1A (a, b, c, and d) 1B (a, b, and c) (*p* ≤ 0.001).

**Biological activity:** The evaluation of the antiproliferative activity of the methanolic extract of the fruit showed that there is a dose-dependent relationship using the doses 1.25, 1.56, 1.85, 2.5, 5, and 10 µg·mL^−1^, where the IC_50_ was 1.85 µg·mL^−1^ (Figure 1B). These data are relevant when compared to the IC_50_ reported for *Sechium edule* var. *amarus silvestrys* of 1170 µg·mL^−1^ (Figure 1A) [16], which is 632 times higher than the IC_50_ of the methanol extract. These same doses were used in an assay with normal lymphocytes, where the decrease in proliferation was not statistically significant (Figure 1B); however, it has already been reported that the normal bone marrow cells of mice are not affected by the extracts [19].

**Figure 2:**
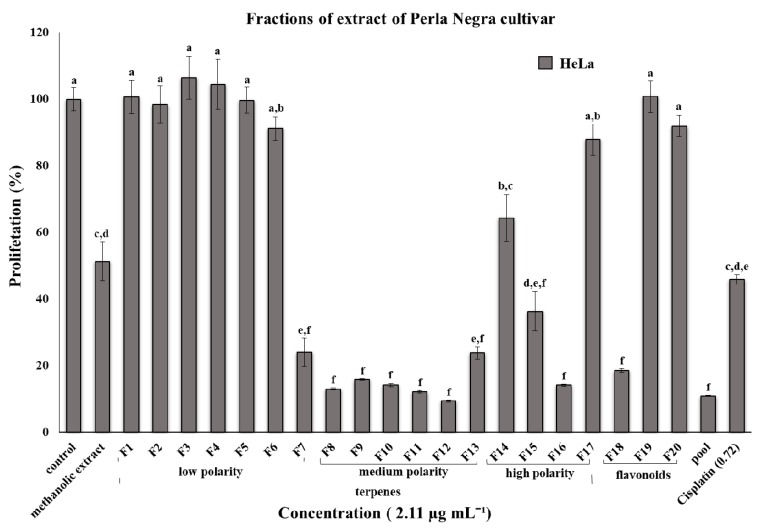
Proliferation assay. The effect of the Perla Negra cultivar fractions on the HeLa cell line. The polarity of fractions is related to the mobile phase of the TLC in the tracking of the terpene compounds. The cells were incubated with all fractions, the methanolic extract, and the pool at the same concentration (2.11 µg·mL^−1^) and Cisplatin (0.72 µg·mL^−1^) for 72 h. Different letters indicate statistical significances (a, b, c, d, e, and f) (*p* ≤ 0.001).

Twenty fractions were obtained: 17 fractions had terpenic compounds, and 3 fractions had phenolic compounds. For the fractions, the evaluated IC_50_ concentration was 2.11 µg·mL^−1^ for each fraction. From the fraction of terpenes, F15 and F7 resulted in 64% and 76% cell proliferation inhibition, respectively, F8-F11, F13, and F16 resulted in 87, 84, 86, 88, 86, and 86% cell proliferation inhibition, respectively, and F12 resulted in 90% cell proliferation inhibition. The F18 fraction, which was related to the flavonoids, showed 82% inhibition (Figure 2). The hexane:ethyl acetate solvent mixture was notable for the chemical fractioning of the extract with the greatest antiproliferative activity, which was attributed to its medium polarity and higher affinity with terpenes.

Additionally, it was observed that when mixing all of the fractions (pool), the antiproliferative percentage of 89% (±0.19) was higher than that for many individual fractions, particularly those from hexane:ethyl acetate (50:50), others fractioned with ethyl acetate:methanol (80:20), and two fractions with flavonoids. The fractioning of the extract with mixtures both of low and high polarity (hexane:ethyl acetate 50:50, ethyl acetate:methanol 20:80) does not reflect the presence of bioactive compounds, such as terpenes, that affect the antiproliferative biological activity. In addition, the value of cell inhibition for the positive control based on Cisplatin™ was lower than ten fractions of the extract.

**Figure 3:**
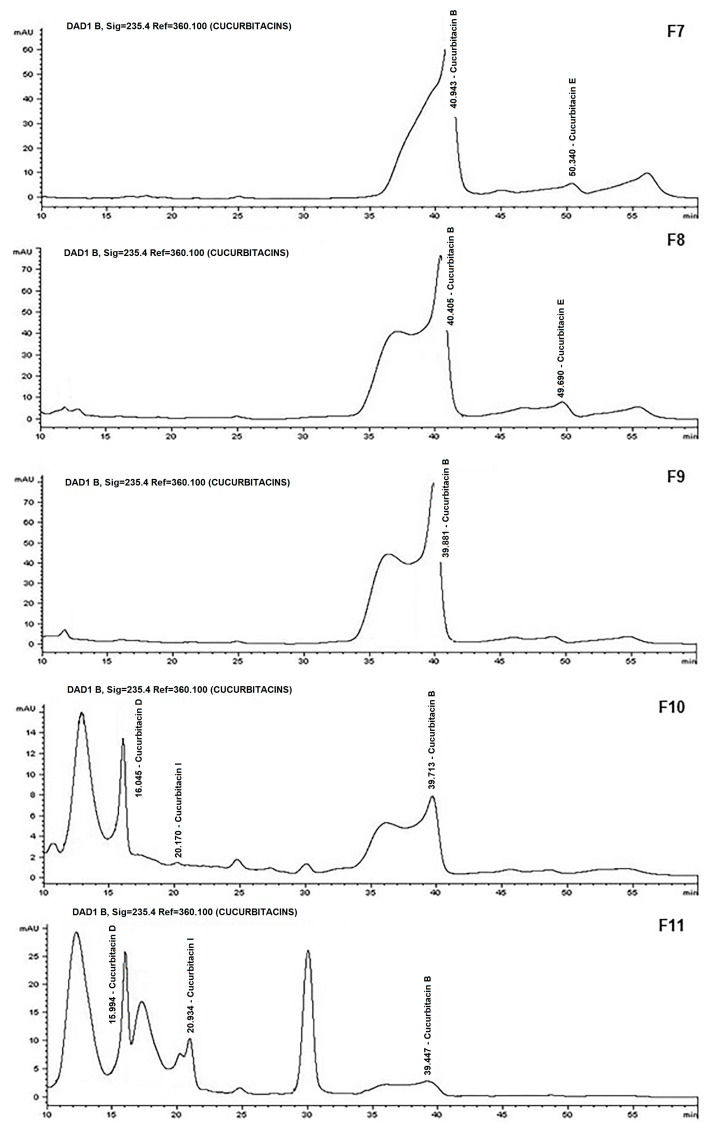

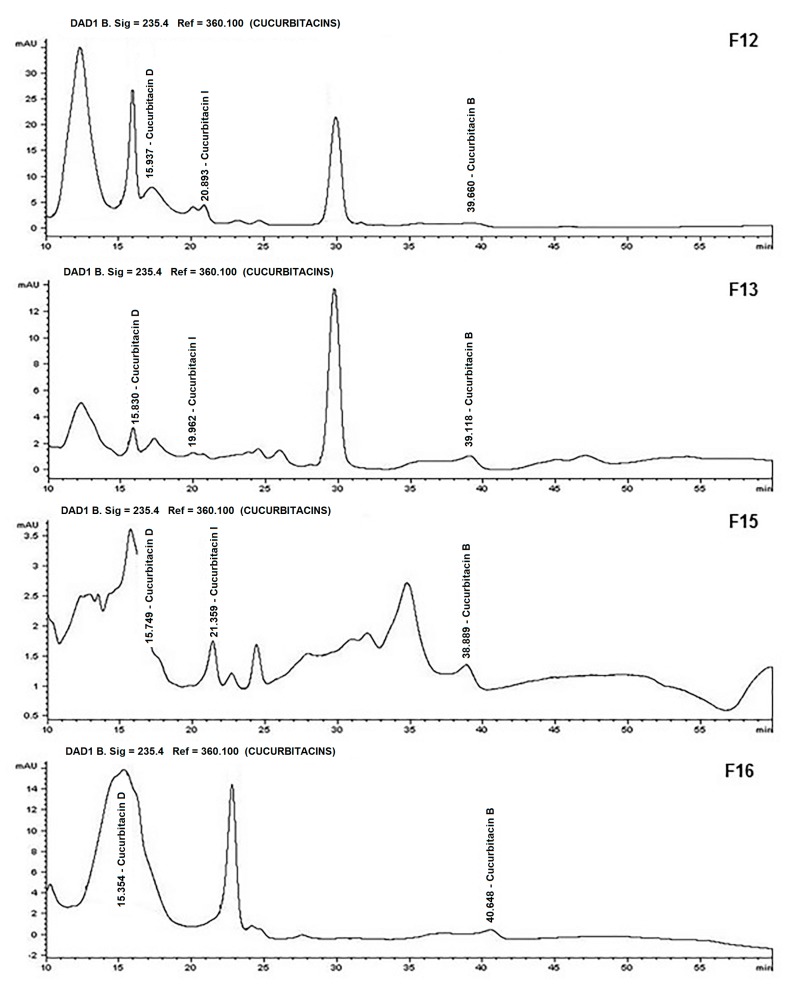
Identifying compounds by HPLC. Chromatograms of the terpenes (cucurbitacins) that showed biological activity, which were obtained from the methanol extract of Perla Negra through column chromatography, where the presence of cucurbitacins and other non-identified compounds is confirmed at 235 nm.

**Identification of compounds:** The identification of compounds with biological activity via TLC showed, based on the value of retention (Rf), that F7, F8, and F9 contained Cucurbitacin B (Rf 0.85) and Cucurbitacin E (Rf 0.86); F10, F11, and F12 contained Cucurbitacins D (Rf 0.76), E, and I (Rf 0.82); and F13 had Cucurbitacin E. For F15 and F16, the cucurbitacin content was not observed with this method. However, the chromatograms obtained via HPLC confirmed the results from TLC, and provided more information about the composition of all of the fractions with biological activity (Figure 3), which additionally detected other compounds different from the standard. These compounds were detected at high concentrations, but their identification was not possible, which suggests that they are cucurbitacins for which the standard was not found or that they are some other terpene with a very similar chemical skeleton to cucurbitacins.

Nine of these fractions had high terpene content obtained via CC from 10 g of crude extract. With respect to the yield of cucurbitacins that could be identified, Cucurbitacin D had the highest concentration, followed by B, E, and I, respectively (Table 1).

**Figure 4:**
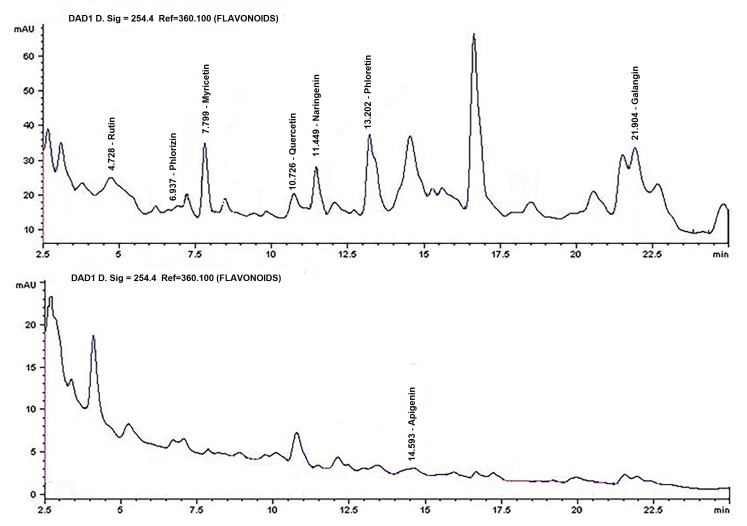
Identifying compounds by HPLC. (**A**) Chromatograms of the flavonoid fractions that showed biological activity, obtained from the methanolic extract of Perla Negra through column chromatography at 254 nm; and (**B**) the presence of Apigenin at 316 nm and unidentified compounds.

The identification of compounds with biological activity from the flavonoid fraction, via HPLC, agreed with the TLC results. Rutin and Quercetin were identified, as well as six more flavonoids, namely, Phlorizidin, Myrecetin, Naringenin, Phloretin, Apigenin and Galangin. Apigenin is a compound that has been previously reported in *Sechium edule* fruits [20]. There are also peaks of compounds that could not be identified (Figure 4A,B).

One of the three fractions stands out because of its flavonoid content. The yield is shown in Table 2, where the Phloretin flavonoid has the highest concentration and Apigenin has lowest concentration. Of note, although Phlorizidin was identified, its concentration could not be calculated, because there were only traces of this compound.

## 4. Discussion

The extracts of fruits from different varietal groups of *Sechium edule* have been reported with terpenes and flavonoids [14,19]; however, the type of metabolite present and its concentration depend on the varietal group, providing different qualities for its various uses and applications [14].

The alcohol extracts of different varietal groups of *Sechium edule*, such as *nigrum spinosum*, *virens levis, amarus silvestrys*, *nigrum xalapensis, albus levis, nigrum levis, albus minor*, and *albus dulcis*, have been reported for their antiproliferative activity against HeLa cells, with IC_50_ values of 930, 500, 1170, 840, 500, 880, 630, and 1510 µg·mL^−1^, respectively [16].

In this study, the methanolic extract of fruits of the *Sechium edule* Perla Negra cultivar had a greater inhibition effect on the HeLa cancer cell line, with an IC_50_ value of 1.85 µg·mL^−1^, which is lower than that of its progenitor *amarus silvestrys* (1170 µg·mL^−1^). This effect is due to the concentration of metabolites they contain, although only the content of cucurbitacins for *amarus silvestrys* (0.1456 g.100 g^−1^) has been reported [15]; Perla Negra cultivar contains 3.2568 g.100 g^−1^ of curcurbitacins and 1.5 g.100 g^−1^ of flavonoids, which provides the biological activity evaluated. This production of metabolites is performed with epigenetic changes that are presented at the time of the interspecific cross for the creation of the Perla Negra cultivar. Interestingly, the methanolic extract of the Perla Negra cultivar does not affect the proliferation of normal human lymphocytes in the same concentration range as the HeLa cells, because the IC_50_ obtained for the lymphocytes is 30.04 µg·mL^−1^. These data suggest that the extract may be selective with the cell type against which it exerts its biological activity, as it has been shown that there are extracts derived from the genus of plants that can affect leukemic lines without inducing cell death to those of the normal bone marrow [19].

Cisplatin™ is a drug widely used against cancer in the clinic, particularly with cervix cancer. Nevertheless, the concentration of Cisplatin™ did not affect the lymphocytes because the concentration used in this study was too low to exert a cytotoxic effect on these cells, and it has been reported that Cisplatin™ at high concentrations does not affect lymphocyte viability [33]. However, Cisplatin™ has been reported as affecting the proliferation of HeLa cells. In this study, we compared Cisplatin™ with the methanolic extract of the Perla Negra cultivar, suggesting that the content of metabolites that make up the extract may act as a cytotoxic agent for cancer cells as does the antineoplastic. It has been reported that the extract from a similar plant induces death by apoptosis to leukemic cells [19], so that this type of death could be present in the HeLa cells. It would be interesting to evaluate Cisplatin™ and the extract synergistically and see if the effect could be potentiated.

The fractions of the methanolic extract of the Perla Negra cultivar obtained by column chromatography showed greater inhibition activity with a concentration of 2.11 µg·mL^−1^ (Figure 2). These effects were not observed in other varietal groups when fractionated. In the varietal group, *nigrum spinosum*, it was observed that the antiproliferative activity decreased significantly when the extract was fractioned [17]. This suggests that not all genotypes of *Sechium edule* have the same biological activity, and that actions taken for genetic improvement to develop cultivars like Perla Negra can improve their yield, chemical composition, and biological activity.

Different authors indicate that the occurrence of epigenetic changes during crossing and hybridization of plants [34,35] generate polymorphisms with new phenotypes and new expression, which are manifested as increases in evolutionary potential [36], favouring responses to the environment, such as abiotic stress [37]. These changes can be partially stable and inheritable in plants [38].

This would explain why the *Sechium edule* Perla Negra cultivar expresses a different potential in terms of the synthesis of secondary metabolites with regard to its progenitors and close genotypes as well as the expression of epigenetic changes in the new cultivar, which would affect its phenotypic expression and response to the environment without directly influencing the genome. In *Sechium edule*, this favours the adaptive specialization to environmental changes, improving the spectrum of plasticity [13,14]. This species has shown a high phenotypic plasticity through its broad intraspecific variation, originating new forms, ecotypes, and varietal groups, and resulting in meta-stable phenotypical novelties attributed to epialleles that can gradually broaden biodiversity [39].

With respect to the biologically-active compounds identified in the methanolic extract of fruit from the *Sechium edule* Perla Negra cultivar, in the terpene fractions were Cucurbitacins D, E, B, and I (Figure 3), and Cucurbitacin D was found at a higher concentration (Table 1). Different studies report on terpenes with antiviral, antibacterial, antihypertensive, anti-hyperglycaemic and anti-parasitic activities as well as their use in central nervous system disorders and cancer treatment [40].

The biological activities of the identified cucurbitacins are as follows. For Cucurbitacin D, the following activities have been observed: antiproliferative activity in vitro in human cells from hepatic carcinoma, endometrial, and ovarian cancer [41,42]; cytotoxic activity on human cell lines from the lungs, colon, prostate, and breast cancer [43]; apoptosis induction in human cells from hepatic carcinoma [41]; and the inhibition of the cell cycle and cell growth in leukaemia [44]. For cucurbitacin I, the following activities have been reported: in vitro and in vivo antiproliferative effects on nasopharynx cancer [45], and cytotoxic activity on HeLa and KB cell lines [43]. Cucurbitacin B has been noted to have a potent inhibitory effect on genes that act in different cell pathways that participate in cellular proliferation [43,46]. Cucurbitacin E affects cell division, generating in vitro disorganization, or breaking, of the cytoskeleton, and inhibiting prostate carcinoma cells [43].

It has been shown that the activity is related to the acetylation of the hydroxyl group or the presence of a double bond, which increases the lipophilicity and toxicity of different cucurbitacins [47]. Cucurbitacins B, E, and I inhibit the signalling transduction and transcription activator JACK/STAT3 [47,48], while Cucurbitacin D inhibits the cell cycle in the G2/M phase [42].

The following were identified for the flavonoid fraction: Rutin, Phlorizidin, Myricetin, Quercetin, Naringenin, Phloretin, Apigenin, and Galangin (Figure 4). Phloretin was found at the highest concentration (Table 2). These compounds have found extensive use in pharmacological areas for different conditions; they have been used as antiallergenic and antidepressant agents for treating thrombosis, diabetes mellitus, rheumatic illnesses, cardiovascular conditions, and gastric ulcers. They also have antibacterial, anti-inflammatory, antioxidant, antiviral, hepatoprotective, and anticancer activity [49,50,51,52,53,54,55,56,57], among other activities.

These compounds can modulate different cell processes, such as proliferation, differentiation, apoptosis, angiogenesis, and metastasis, and can thus interfere with the development of cancer. They are also efficient in eliminating oxidant molecules and various free radicals, which can be implicated in promoting tumours via DNA damage [54,56].

In various in vitro studies, it was confirmed that Quercetin has inhibitory effects on different cell lines, such as colon, breast, ovary, and gastrointestinal cancer [51], as well as on lymphoma and leukaemia, cervix, stomach, ovary, and pancreas cancer cells lines, independent of polyhydroxyilation [53]. The inhibition of melanoma growth by this flavonoid and Apigenin is also reported [58]. Flavonoids like Fisetin, Apigenin, and Luteolin are inhibitors of cell proliferation [50]. With regard to the other flavonoids identified, no report was found in the reviewed literature that indicated an effect on other cell lines; therefore, this study contributes new information about flavonoids, which have antiproliferative activity against the HeLa cancer cell line with an IC_50_ of 2.11 µg·mL^−1^, for the fraction from which they were identified.

The biological activity of flavonoids depends the chemical structure, degree of hydroxylation and polymerization, and other substitutions and conjugations. The major molecular mechanisms of action of flavonoids are as follows: the downregulation of the mutant p53 protein, cell cycle arrest, tyrosine kinase inhibition, inhibition of heat shock proteins, estrogen receptor binding capacity, and the inhibition of Ras protein expression [57].

Thus, the biological effect shown by the Perla Negra cultivar suggests that it could be investigated as a potential anticancer agent because its IC_50_ is lower than the maximum value of 20 µg·mL^−1^ established by the United States National Cancer Institute [58], besides not affecting the proliferation of normal cells (lymphocytes). Therefore, it is essential to assess the toxicity of the extract in healthy mice to determine its safety as a potential medicinal agent. It would also be important to know the bioavailability of the extract compounds, and even their metabolism on in vivo models, before their extrapolation as an antineoplastic agent.

## 5. Conclusions

The presence of the terpene and flavonoid groups in the methanolic extract of the Perla Negra cultivar was confirmed. The Perla Negra cultivar inherited biological activity from its parents, which was likely reinforced by epigenetic changes. The methanol extract had an IC_50_ that was 632 times lower than that reported for its parent. The extraction and fractioning of the plant extract with medium polarity agents was more efficient, and showed higher antiproliferative activity. Ten fractions stood out with IC_50_ values lower than the crude extract. The compounds identified in the fractions with biological activity were Cucurbitacins D, E, B, and I for the terpene fractions and Rutin, Phlorizidin, Myricetin, Quercetin, Naringenin, Phloretin, Apigenin, and Galangin for the flavonoid fractions. Therefore, the antiproliferative effect shown by the Perla Negra cultivar opens the possibility of being investigated as a possible anticancer agent.

## Figures and Tables

**Table 1:** Cucurbitacin yield (µg·mg^−1^) of nine fractions of the methanolic extract from the fruits of the *Sechium edule* Perla Negra cultivar that had biological activity against the HeLa cancer cell line.

FRACTION (mg)	YIELD (%)	Cu B (µg)	Cu D (µg)	Cu E (µg)	Cu I (µg)
F7	1.59	29.61	0	42.39	0
F8	1.44	48.16	0	53.84	0
F9	0.74	53.12	0	0	0
F10	1.34	5.52	75.09	0	0
F11	1.81	0	108.60	1.53	30.40
F12	1.49	0	134.67	0.46	8.92
F13	1.44	0.29	7.06	0	0
F15	1.28	0	16.13	0	1.05
F16	3.94	0.30	594.39	0	0
TOTAL	15.07	137.00	935.94	98.21	40.37

Cu B: Cucurbitacin B; Cu D: Cucurbitacin D; Cu E: Cucurbitacin E; Cu I: Cucurbitacin I.

**Table 2:** Flavonoid yield (µg·mg^−1^) found in F18 obtained from the methanolic extract of the fruits of *Sechium edule* Perla Negra cultivar that had biological activity against the HeLa cancer cell line.

Fraction (mg)	Yield (%)	Rutin (µg)	Miricetin (µg)	Quercetin (µg)	Naringenin (µg)	Phloretin (µg)	Galangin (µg)	Apigenin (µg)
F18	12.07	0.34	1.85	0.25	2.26	4.72	0.43	0.18
